# Combination therapy of Tocilizumab and steroid for management of COVID-19 associated cytokine release syndrome: A single center experience from Pune, Western India

**DOI:** 10.1097/MD.0000000000026705

**Published:** 2021-07-23

**Authors:** Ameet Dravid, Reema Kashiva, Zafer Khan, Danish Memon, Aparna Kodre, Prashant Potdar, Milind Mane, Rakesh Borse, Vishal Pawar, Dattatraya Patil, Debashis Banerjee, Kailas Bhoite, Reshma Pharande, Suraj Kalyani, Prathamesh Raut, Madhura Bapte, Anshul Mehta, M. Sateesh Reddy, Krushnadas Bhayani, S. S. Laxmi, P. D. Vishnu, Shipra Srivastava, Shubham Khandelwal, Sailee More, Rohit Shinde, Mohit Pawar, Amol Harshe, Sagar Kadam, Uma Mahajan, Gaurav Joshi, Dilip Mane

**Affiliations:** a Department of Infectious Diseases and HIV/AIDS, Noble hospital and Research Centre, Pune, Maharashtra, India; b Department of Medicine, Noble hospital and Research Centre, Pune, Maharashtra, India; c Department of Critical Care Medicine, Noble hospital and Research Centre, Pune, Maharashtra, India; d Department of Pathology, Noble hospital and Research Centre, Pune, Maharashtra, India; e Department of Radiology, Noble hospital and Research Centre, Pune, Maharashtra, India; f VMK Diagnostics Private Limited, Pune, Maharashtra, India.; g Independent statistical consultant, Chicago.

**Keywords:** COVID-19, cytokine release syndrome, infectious complications, intensive care, mechanical ventilation, steroids, Tocilizumab

## Abstract

Cytokine release syndrome (CRS) or cytokine storm is thought to be the cause of inflammatory lung damage, worsening pneumonia and death in patients with COVID-19. Steroids (Methylprednislone or Dexamethasone) and Tocilizumab (TCZ), an interleukin-6 receptor antagonist, are approved for treatment of CRS in India. The aim of this study was to evaluate the efficacy and safety of combination therapy of TCZ and steroid in COVID-19 associated CRS.

This retrospective cohort study was conducted at Noble hospital and Research Centre (NHRC), Pune, India between April 2 and November 2, 2020. All patients administered TCZ and steroids during this period were included. The primary endpoint was incidence of all cause mortality. Secondary outcomes studied were need for mechanical ventilation and incidence of systemic and infectious complications. Baseline and time dependent risk factors significantly associated with death were identified by Relative risk estimation.

Out of 2831 admitted patients, 515 (24.3% females) were administered TCZ and steroids. There were 135 deaths (26.2%), while 380 patients (73.8%) had clinical improvement. Mechanical ventilation was required in 242 (47%) patients. Of these, 44.2% (107/242) recovered and were weaned off the ventilator. Thirty seven percent patients were managed in wards and did not need intensive care unit (ICU) admission. Infectious complications like hospital acquired pneumonia, blood stream bacterial and fungal infections were observed in 2.13%, 2.13% and 0.06% patients respectively. Age ≥ 60 years (*P* = .014), presence of co-morbidities like hypertension (*P* = .011), IL-6 ≥ 100 pg/ml (*P* = .002), D-dimer ≥ 1000 ng/ml (*P* < .0001), CT severity index ≥ 18 (*P* < .0001) and systemic complications like lung fibrosis (*P* = .019), cardiac arrhythmia (*P* < .0001), hypotension (*P* < .0001) and encephalopathy (*P* < .0001) were associated with increased risk of death.

Combination therapy of TCZ and steroids is likely to be safe and effective in management of COVID-19 associated cytokine release syndrome. Efficacy of this anti-inflammatory combination therapy needs to be validated in randomized controlled trials.

## 1 Introduction

In December 2019, Wuhan city, the capital of Hubei province in China, became the centre of an outbreak of viral pneumonia caused by a novel beta Coronavirus. It was named severe acute respiratory syndrome coronavirus 2 (SARS-CoV-2) due to its sequence homology with SARS-COV-1.^[1]^ The disease caused by SARS-COV-2 was later designated Coronavirus disease 2019 (COVID-19) in February 2020, by World health organization (WHO).^[2]^ COVID-19 spread rapidly worldwide and India was no exception. By November 2, 2020 there have been more than 9 million infections and 0.14 million deaths due to COVID-19 in India.^[3]^ Severe and critical disease develops in approximately 15% and 5% of COVID-19 patients. In these patients, pneumonia leads to acute respiratory distress syndrome (ARDS) that could need invasive mechanical ventilation.^[1,4–7]^ Critically ill COVID-19 patients have a mortality rate ranging from 35 to 62%.^[8–10]^

### 1.1 Role of host inflammatory response in severe COVID-19

COVID-19 is characterized by 2 phases; viral replication phase and the host inflammatory response phase.^[11]^ Host inflammatory response phase is usually seen 7 days after symptom onset. SARS-CoV-2 replicates within the pulmonary tissue, activates innate immune response, leading to production of cytokines (Interleukin-1 beta (IL-1B), Interleukin-6 (IL-6) and Tumor necrosis factor (TNF)) by alveolar macrophages and recruitment of adaptive immunity cells. The transition between innate and adaptive immune responses is critical for the clinical trajectory of SARS-CoV-2 infection.^[11–15]^ Adaptive immune response controlled by immune regulatory cells can be a protective immune response or a dysregulated and exacerbated inflammatory response.^[12]^ Amongst severe and critically ill COVID-19 patients, there is a dysregulated pulmonary and systemic immune response. This dysregulated immune response also known as cytokine release syndrome (CRS) or cytokine storm is characterized by elevation in systemic inflammatory markers (C-reactive protein (CRP), ferritin, lactate dehydrogenase (LDH) and D-dimer) and aberrant pro-inflammatory cytokine secretion (IL-6, soluble IL-2 receptor [IL-2R], IL-10, TNF-α) by pulmonary alveolar macrophages. It is also accompanied by depleted adaptive immune response in the form of lymphopenia (decline in CD4+ T cell, CD8+ T cell, Natural killer cell but not in B cell subset) and decreased Interferon gamma (IFN-γ) expression in CD4+ T cells (CD4+ T cell dysfunction).^[12–15]^ Cytokine storm leads to destruction of alveolar epithelial cells, increased pulmonary vascular permeability, worsening pneumonia, increased risk of thrombosis and progression to acute respiratory distress syndrome (ARDS).^[15]^ Rising levels of interleukin-6 (IL-6) in severe COVID-19 have been associated with increased likelihood of ARDS, mechanical ventilation and mortality.^[16–19]^

### 1.2 Role of immunomodulatory drugs in tackling cytokine release syndrome

Steroids, namely Dexamethasone and Methylprednisolone have been extensively used to resolve hyperinflammation and inflammatory lung damage in COVID-19.^[20–23]^ After the publication of RECOVERY trial, Dexamethasone was approved by World health organization (WHO) as an immunomodulatory drug for use in hospitalized COVID-19 patients who require oxygen. The benefit of Dexamethasone was greatest for patients who were receiving invasive mechanical ventilation.^[21]^ In patients with severe COVID-19, Methylprednisolone therapy was associated with reduced rate of ICU transfer, reduced rate of progression to mechanical ventilation and higher probability of extubation in patients already on mechanical ventilation.^[22,23]^ However, steroids alone might not be able to tackle cytokine storm in all patients. Other Immunomodulatory drugs such as selective cytokine inhibitors could be of incremental benefit to suppress the hyperinflammation if used in combination with steroids.

Tocilizumab (TCZ) is a recombinant humanized monoclonal interleukin-6 receptor (IL-6R) antibody of the IgG1 subtype. TCZ specifically binds and inhibits soluble and membrane-bound IL-6 receptors (sIL-6R and mIL-6R) and terminates downstream intracellular signal transduction.^[24,25]^ It has been used for the treatment of rheumatoid arthritis,^[26]^ systemic juvenile idiopathic arthritis,^[27]^ Castleman disease,^[28]^ Crohn's disease^[29]^ and cytokine release syndrome (CRS) caused by chimeric antigen receptor T-cell (CAR-T) immunotherapy.^[30]^ Indian council of Medical research (ICMR) guidelines published by Government of India for guidance of physicians, have included TCZ as investigational therapy for off label use in patients with moderate disease with progressively increasing oxygen requirements and in mechanically ventilated patients not improving despite use of steroids.^[31]^

This recommendation stems from multiple retrospective and small prospective studies,^[32–58]^ that have suggested strong clinical benefits due to use of TCZ in CRS in form of reduced risk of invasive mechanical ventilation or death in patients. However data from randomized controlled trials (RCT) has shown disappointing results with evidence, if at all, of modest efficacy.^[59–63]^ RCT's have shown that TCZ reduces need for ICU admission and noninvasive and invasive ventilation in patients with severe COVID-19.^[61,62]^ However, none of them have reported mortality benefit. Out of the 5 RCT's, only the EMPACTA trial^[62]^ used concomitant TCZ and steroids in management of CRS. As a result, more evidence regarding positioning of TCZ as an immunomodulatory therapy in COVID-19 needs to be published. Data from resource limited settings like India regarding use of TCZ and steroids in management of CRS has also been scarce.^[64,65]^

## 2 Aims and objectives

The aim of this single center retrospective cohort study conducted in Pune, India was to estimate the efficacy and safety of combination therapy of TCZ and steroid in management of COVID-19 associated CRS.

## 3 Methods

### 3.1 Study setting

This study was conducted at Noble hospital and Research Centre (NHRC), Pune, Western India. NHRC is a tertiary level private hospital designated for clinical management of COVID-19 patients in Pune since March 23, 2020. Pune is located in the state of Maharashtra, Western India and was one of the epicenters of COVID-19 epidemic in India. As of November 2, 2020, Maharashtra state has reported more than 1.71 million cases of COVID-19 and more than 45,000 deaths.^[3]^ Till November 2, 2020, NHRC has admitted 2831 COVID-19 patients.

NHRC provides clinical care, diagnostic and treatment services to COVID-19 patients at a subsidized cost. Patients are referred from primary care physicians, private practitioners of alternative medical systems, primary level COVID care centers (both government run and private owned) and other tertiary level hospitals. Data of all hospitalized COVID-19 patients is entered into an electronic database (Lifeline electronic database, Manorama infosystems, Kolhapur, India). Data was obtained from electronic health record of each individual by manual abstraction. It included hospitalization dates, demographics, co-morbidities, clinical examination data, laboratory data (including inflammatory markers), microbiology reports and imaging reports (X-ray chest and high-resolution computerized tomography scan (HRCT chest)).

### 3.2 Study population

Patients were eligible for inclusion in this analysis if they were admitted to NHRC between April 2, 2020 and November 2, 2020 and were administered TCZ. Criteria for prescribing TCZ were developed by the Department of Infectious Diseases and Department of Critical care medicine. Patients were administered TCZ if they satisfied following **inclusion criteria for hyperinflammation or CRS:**

(1) Reverse-transcriptase polymerase chain reaction (RT-PCR) test positive for SARS-CoV-2 RNA or positive COVID antibody test.
(2) Lung Imaging: Moderate or severe pneumonia on High resolution Computerized tomography scan (HRCT) of chest (CT severity index > = 8^[66]^) or X-ray chest showing evidence of pneumonia.
(3) Day 7 to 14 since onset of symptoms.
(4) Rapidly worsening respiratory status despite use of steroids and antiviral drugs: Hypoxia (room air oxygen saturation (SPO2) < 90 mm Hg) and tachypnea (respiratory rate > 30 per minute) at rest or after minimal exertion which requires supplemental oxygen. OR
(5) Rapidly worsening respiratory status despite use of steroids and antiviral drugs: Requirement of noninvasive or invasive ventilation to mitigate hypoxia and tachypnea. OR
(6) PaO2/FiO2 ratio (ratio of arterial oxygen partial pressure (PaO2 in mmHg) to fractional inspired oxygen (FiO2) of less than 300 on room air.
(7) Elevated inflammatory markers: IL-6 (> 100 pg/ml or 5-fold increase from prior level) or one out of D-dimer (> 1000 ng/ml), Ferritin (> 1000 ng/ml) and CRP (> 10 mg/ml) being elevated.

**Exclusion criteria:** Following patients were not prescribed TCZ during hospital admission or were excluded from the analysis.

(1) Pregnant females.
(2) Patients having active systemic infections (bacterial/fungal), active tuberculosis and active hepatitis B/C co-infection.
(3) History of diverticulitis or inflammatory bowel disease.
(4) Patients who refused TCZ therapy for management of CRS.
(5) Patients administered TCZ in another institute prior to transfer to NHRC.

Tocilizumab was given intravenously at 8 mg/kg bodyweight (up to a maximum of 800 mg in two infusions, 24 h apart). Additional doses were given at the discretion of infectious disease and critical care physician if it was thought that additional dosing would be needed for morbidly obese patients (body weight > 100 kg) or to reduce persistent hyperinflammation. Patients or their immediate family members signed an informed consent form prior to TCZ administration. The language in the consent form was non-prescriptive, saying that TCZ can be used off-label in patients with COVID-19 induced hyperinflammation as per ICMR guidelines. The consent form cautions that the evidence for benefit is modest; a risk of infectious complications exists but in view of limited treatment options in patients with severe pneumonia, therapy can be considered. The cost of TCZ (approximately 500 dollars for 1 vial of 400 mg) was borne by the patient as an out of pocket expense or by third party reimbursement via medical health insurance.

### 3.3 Data collection

For all patients administered TCZ, we scrutinized inpatient case files until hospital discharge, death, or December 2, 2020—the date on which the database was locked—whichever happened first. Number of TCZ doses given, type of steroid and dose of steroid given and other concomitant medication prescribed to patient were noted. Time to TCZ administration since onset of symptoms and time to TCZ therapy since hospital admission was calculated. Oxygenation and ventilation outcomes in patients were noted. Systemic complications (including infectious complications) developing in patients during hospital admission were also noted. All patients who died during hospital admission were identified and a death audit to look for complications and cause of death was undertaken. Ordinal scale for COVID-19 severity was noted for all patients at hospital admission, during hospital stay and at discharge or death. The 8 level ordinal scale is as follows: 1 = ambulatory and no restriction of activities; 2 = ambulatory and restriction of activities due to use of home oxygen therapy or complications; 3 = Hospitalized but no oxygen therapy; 4 = Hospitalized with oxygen therapy by nasal prongs; 5 = Hospitalized with oxygen therapy by Non re-breathing mask; 6 = Hospitalized with severe disease and on high flow nasal oxygen (HFNO) or Noninvasive ventilation; 7 = Hospitalized with severe disease and on invasive mechanical ventilation; 8 = Death.

All patients who recovered, got discharged and had outpatient follow-up visit at 15 and 30 days after discharge were identified. Their outpatient follow-up visits were traced from electronic database to look for delayed complications. There was no control group in our study as all patients with suspicion of hyperinflammation or CRS ended up getting TCZ and steroid.

### 3.4 Concomitant medications given for COVID-19 therapy

NHRC follows the ICMR guidelines published by Government of India for COVID-19 management.^[31]^ These guidelines get updated from time to time. All hypoxic COVID-19 patients who received TCZ were already receiving antiviral agents, intravenous steroids (Dexamethasone 6 mg per day or Methylprednisolone 40 mg BD) and systemic anticoagulation (Low molecular weight heparin (Enoxaparin) or Unfractionated Heparin) as a standard of care. Intravenous steroids were continued for a maximum of 10 days followed by shift to oral Prednisolone in tapering doses over next 10 days. Hydroxychloroquine and Lopinavir/ritonavir were initially recommended as standard antiviral therapy at NHRC. Once clinical trial data about lack of efficacy and toxicity was published, their use as antivirals was discontinued.^[67–69]^ Remdesvir was used as antiviral of choice at our institute since July 26, 2020 and was administered to all patients presenting with moderate or severe pneumonia.^[70]^ Convalescent plasma therapy (CPT) was also used in a subset of patients as an antiviral agent. Adjunctive antibiotic and antifungal therapy in patients to prevent bacterial and fungal super-infections was decided by the infectious disease physician. After administration of TCZ, anti-fibrotic agents like Pirfenidone (daily dosage ranging from 800 to 2000 mg per day) and Nintedanib (daily dosage of 150 mg twice a day) were added to the treatment protocol for patients suspected of developing lung fibrosis.

### 3.5 Outcomes

#### 3.5.1 Primary endpoint

Deaths in the cohort after TCZ and steroid administration.

#### 3.5.2 Secondary outcomes

(A) Number of patients who received noninvasive or invasive ventilation: Patients who required mechanical ventilation (noninvasive or invasive) in our cohort were identified. NHRC adopted the delayed intubation and delayed invasive mechanical ventilation (IMV) policy for patients with COVID-19 associated ARDS. Patients not maintaining arterial oxygen saturation (SPO2) > 90% on supplemental oxygen (Non re-breathing mask at 15 liters/minute) were initially started on noninvasive ventilation (NIV: High flow nasal oxygen (HFNO) or Bi-level positive airway pressure (BIPAP)). The indications for invasive mechanical ventilation (IMV) in our hospital was respiratory failure on NIV, defined by any of the following criteria:

1. respiratory rate of 40 or more breaths per minute;
2. respiratory distress with activation of accessory respiratory muscles;
3. the need for fractional inspired oxygen (FiO2) of 100% to maintain an oxygen saturation (SPO2) level of 90%, or a PaO2/FiO2 ratio of less than 100;
4. Neurological deterioration (altered consciousness with a Glasgow Coma Scale score of 10 or lower) on treatment.

(B) Number of patients who could be weaned off NIV or IMV and discharged from hospital.

(C) Improvement in 8 point ordinal scale reflecting declining disease severity.

(D) Incidence of infectious complications (bacterial or fungal super-infections): Patients developing fever (Temperature > 38.3 degrees Celsius), productive cough, leucocytosis (WBC count > 15,000 cells/mm^3^ with predominance of neutrophils and shift to left) or increased serum procalcitonin despite adequate antibiotic and antifungal therapy were investigated for super-infections. All positive blood and respiratory cultures (bacterial and fungal culture) were assessed by an Infectious Diseases physician to decide infection versus colonization. Infections were included if they occurred > 48 h after TCZ administration.

(E) Incidence of systemic complications like cardiovascular (acute myocardial infarction, congestive cardiac failure, arrhythmia and myocarditis), respiratory (lung fibrosis and pulmonary embolism), neurologic (stroke, encephalopathy,^[71]^ meningo–encephalitis, transverse myelitis and Guillaine Barre syndrome (GBS)), gastrointestinal (intestinal perforation, gastroenteritis and abdominal blood vessel thrombosis), hepatic (hepatitis) and renal complications (acute kidney injury^[72]^) developing during hospital admission were estimated.

The use of database for clinical research was approved by the institutional review board (IRB) of Noble hospital and Research Centre, Pune, India.

### 3.6 Statistical methods

Continuous variables were summarized using median and inter-quartile range (IQR), while categorical variables were summarized using frequency and percentages. Continuous variables were compared using a Mann–Whitney *U* test. Categorical variables were compared using Chi-square test, Proportion test and Fishers’ exact test. Baseline and time dependent risk factors significantly associated with death were identified by Relative risk estimation. Baseline risk factors included were age (< 60 years or ≥ 60 years), gender, co-morbidities like diabetes, hypertension, ischemic heart disease and chronic kidney disease and baseline investigations like IL-6 (≥ 100 versus < 100 pg/ml), absolute lymphocyte count (< 1000 versus ≥ 1000 cells/mm^3^), D-dimer (≥ 1000 versus < 1000 ng/ml) and CT severity index (≥ 18 versus < 18). Time dependent risk factors included systemic complications like lung fibrosis, arrhythmia, hypotension, new onset or worsening thrombocytopenia, hepatitis, acute kidney injury and encephalopathy. The *P* value ≤ .05 was considered as statistically significant. All data was analyzed by SPSS version 12.0.

## 4 Results

### 4.1 Baseline demographic data

Of the total 2831 COVID-19 patients admitted in NHRC, 522 were administered TCZ. Seven patients were given TCZ therapy prior to transfer to NHRC and hence were excluded from the analysis (Number of patients included in final analysis – 515). Baseline demographic data, preexisting co-morbidities, presenting symptoms in patients at admission and investigations performed prior to TCZ therapy are enumerated in Tables 1 and 2. Median age of the cohort was 57 (IQR: 46.5, 66) years and it included 24.3% females. Two hundred and twenty-two (43.1%) patients were ≥ 60 years of age. Diabetes mellitus (45.4%), Hypertension (48.3%), Ischemic heart disease (13.6%), Chronic kidney disease (7.8%) and Obesity (Body mass index > 30 kg/m^2^, 9.5%) were the commonest co-morbidities seen in our cohort (Table 1). Fever (81%), dry cough (77%), dyspnea on exertion (81%) and bodyache or myalgia (49%) were the commonest symptoms seen in patients (Table 1). Out of 373 patients who performed HRCT of Chest (GE Optima, 128 slice CT scanner), CT severity index prior to TCZ therapy indicated moderate disease (CT severity index: 8–14) in 37.5% (140/373) and severe disease (CT severity index: 15–25) in 62.5% (233/373) of patients. Three hundred and eighty seven (387/515, 75.1%) patients had arterial blood gas analysis performed prior to TCZ therapy. PaO2/FiO2 ratio was in the range of 200–300, 100–200 and < 100 amongst 65 (16.8%), 182 (47%) and 140 (36.2%) individuals respectively.

**Table 1:** Baseline characteristics of patients in the cohort.

Co-morbidities	Total cohort n = 515 (%)	Presenting symptoms	n (%)
Diabetes mellitus	234 (45.4%)	Fever	419 (81%)
Hypertension	249 (48.3%)	Dry cough	398 (77%)
Ischemic heart disease	70 (13.6%)	Cough with expectoration	36 (7%)
Chronic kidney disease	40 (7.8%)	Hemoptysis	12 (2%)
Lung disease	22 (4.3%)	Myalgia or Bodyache	254 (49%)
Liver disease (Liver cirrhosis)	7 (1.4%)	Chills	76 (15%)
Stroke	5 (1%)	Headache	58 (11%)
Other Neurologic comorbidities	10 (1.9%)	Loss of appetite	73 (14%)
History of cancer	6 (1.1%)	Dyspnoea at rest or on exertion	419 (81%)
Past history of pulmonary Tuberculosis	5 (1%)	Anosmia	20 (4%)
HIV infection	3 (0.6%)	Dysgeusia	59 (11%)
Obesity	49 (9.5%)	Diarrhea	44 (9%)
Thyroid disorder	41 (7.9%)	Vomiting	36 (7%)
Rheumatologic disorders	10 (1.9%)	Abdominal pain	11 (2%)
0 co-morbidities	142 (27.6%)	Chest pain/Chest tightness	19 (4%)
1 comorbidity	144 (28%)	Upper respiratory tract symptoms	69 (13%)
2 comorbidity	134 (26%)	Giddiness	22 (4%)
3 or more co-morbidities	95 (18.4%)		

**Lung disease:** Chronic obstructive pulmonary disease, Asthma, Interstitial lung disease, Obstructive sleep apnea. **Other neurologic comorbidities:** Parkinson's disease, Alzheimer's disease, Seizure disorder, Cerebral palsy, Poliomyelitis, Psychiatric disorders; **Rheumatologic co-morbidities:** Rheumatoid arthritis, Gouty arthritis, Psoriasis, Systemic lupus erythematosus, Vasculitis; **Thyroid disease:** Hypothyroidism, Hyperthyroidism. **Upper respiratory tract symptoms:** Runny nose; Sore throat; Nasal congestion; Sneezing.

**Table 2:** Demographic features and baseline investigations in patients administered Tocilizumab.

Investigations	All patients (n = 515) Median, IQR	Patients who recovered and were discharged (n = 380), Median, IQR	Patients who died (n = 135), Median, IQR	*P* value
Age	57 (46.5,66)	55 (45,64)	62 (53,69)	< .0001
Gender: Male	390 (75.7%)	293 (77.1%)	97 (71.9%)	.226
Gender: Female	125 (24.3%)	87 (22.9%)	38 (28.1%)	.226
Any co-morbidity	373 (72.4%)	260 (69.7%)	113 (83.7%)	.004
Diabetes mellitus	234 (45%)	167 (44%)	67 (49%)	.253
Hypertension	249 (48%)	171 (45%)	78 (58%)	.01
Ischemic heart disease	70 (14%)	46 (12%)	24 (18%)	.097
Chronic kidney disease	40 (8%)	23 (6%)	17 (13%)	.016
Hemoglobin (g/dl)	13.1 (11.6,14.5)	13.2 (11.7,14.5)	12.9 (11.2,14)	.092
White blood cell count (cells/mm3)	6900 (5000,10,300)	6500 (4800, 9600)	8400 (5575, 12,100)	< .0001
Absolute lymphocyte count (cells/mm3)	1071 (760,1488)	1089 (795, 1519)	1001 (659, 1440)	.114
Platelet count (cells/mm3)	207,000 (163,000, 268,000)	207,000 (161,000, 268,500)	208,000 (167,000, 268,000)	.477
SGPT (U/L)	35 (22, 51)	34 (23,51)	37 (22,53)	.952
SGOT (U/L)	43 (30,64)	42 (31,61)	47 (29,73)	.219
Blood Urea (mg/dl)	31 (22,43)	29 (20,39)	37 (25,55)	< .0001
Creatinine (mg/dl)	1.08 (0.93,1.19)	1.07 (0.93,1.19)	1.12 (0.94,1.31)	.105
IL-6 Normal range: 0–7 pg/ml	69.83 (32.53, 117.00)	60.17 (31.28, 105.85)	87.20 (48.00, 161.90)	< .0001
CRP Normal range: 0–6 mg/L	54.92 (30.36, 96.29)	53.42 (30.99, 85.56)	62.74 (27.29, 110.08)	.190
D dimmer Normal range: < 252 ng/ml	344.0 (256.0, 814.5)	318.5 (251.0, 568.0)	593.0 (316.0, 1200)	< .0001
Ferritin Normal range: 15–150 ng/ml	494.45 (238.30, 976.80)	448.50 (219.60, 855.10)	687.40 (305.90,1274.00)	.002
Procalcitonin Normal range: < 0.05 ng/ml	0.21 (0.17, 0.28)	0.21 (0.17, 0.28)	0.22 (0.17, 0.28)	.391
CT severity index	16 (12,18)	15 (12,17)	18 (16, 20)	< .0001

SGOT = Serum glutamate ornithyl transferase, SGPT = Serum glutamate pyruvate transferase, IL-6 = Interleukin-6, CRP = C reactive protein, CT = Computerized tomography scans.

### 4.2 Efficacy of Tocilizumab and Steroid combination therapy

Total cumulative dose of TCZ administered was 400 mg, 800 mg and 1200 mg in 47.2% (243/515), 48% (247/515) and 4.8% (25/515) patients respectively. Median time to administering TCZ after onset of symptoms was 9 days (IQR: 7, 11) and median time to TCZ administration after hospital admission was 2 days (IQR: 2, 4). Overall 87.2%, 90% and 94.4% patients were prescribed Remdesvir, Methylprednisolone and low molecular weight or conventional heparin along with TCZ (Table 3). Intensive care unit (ICU) admission was needed in 63.6% (327/515) of patients while 36.4% (187/515) patients recovered in isolation wards. Two hundred and seventy-three patients (273/515, 53%) required only supplemental oxygen prior to recovery (improvement in ordinal scale from 4 or 5 to 1). Two hundred and forty-two patients (47%) needed non-invasive (NIV) and/or invasive mechanical ventilation (IMV) during admission, of which 44.2% (107/242) recovered and were weaned off the ventilator (improvement in ordinal scale from 6 or 7 to 1). Median time to hospital discharge or death in our cohort was 13 days (IQR: 10, 17). Among patients admitted in ICU, median duration of ICU stay was 10 days (IQR: 7, 16). In patients needing mechanical ventilation (NIV or IMV), median duration of ventilation was 10 days (IQR: 6, 13). Twenty-one (4.1%) patients were administered hemodialysis while 105 (20.4%) needed vasopressor agents like noradrenaline and vasopressin for hypotension (Table 3).

**Table 3:** Concomitant drugs administered along with Tocilizumab.

Concomitant Medication (N = 515)	N	%
Remdesvir	449	87%
Methylprednisolone	463	90%
Dexamethasone	52	10%
Enoxaparin/ Conventional Heparin	486	94%
Hydroxychloroquine	16	3.1%
Lopinavir-ritonavir	231	45%
Convalescent plasma therapy	231	45%
Methylene blue	153	30%
Pirfenidone	239	46.41%
Nintedanib	81	15.73%
Piperacillin–Tazobactum	154	30%
Meropenem	359	70%
Teicoplanin	324	63%
Fluconazole	382	74.2%
Caspofungin	99	19.2%
Noradrenaline	105	20.4%
Vasopressin	41	8%
Hemodialysis	21	4%

### 4.3 Systemic and Infectious complications

Systemic complications (fatal and nonfatal) observed in patients in our cohort are enumerated in Table 4. Septic shock (persistent hypotension requiring vasopressors to maintain systolic blood pressure > 100 mm Hg and a serum lactate level greater than 2 mmol/L) was noted in 58 (11.3%) patients, of whom 4 recovered after treatment. Infectious complications like hospital or ventilator associated pneumonia (HAP/VAP), blood stream bacterial infections and blood stream fungal infections were seen in 2.13% (11/515), 2.13% (11/515) and 0.06% (3/515) patients respectively. Methicillin resistant *Staphylococcus aureus* (MRSA) was the commonest bacteria (6/11 cases) and *Candida albicans* was the commonest fungus (3/3 cases) isolated in blood culture. Multidrug resistant Gram-negative bacilli (*Pseudomonas Aeruginosa* (3/11), *Acinetobacter Baumanii* (3/11) and *Klebsiella pneumonia* (2/11)) were the commonest causative agents of HAP or VAP.

**Table 4:** Systemic complications in patients after administration of Tocilizumab.

Type of complication	Total cohort n = 515 (%)	Patients who recovered n = 380 (%)	Patients who died n = 135 (%)	*P* value
Subcutaneous emphysema/ Pneumothorax	26 (5.1%)	1 (0.3%)	25 (18.5%)	<.0001
Hemoptysis	23 (4.5%)	18 (4.7%)	5 (3.7%)	.628
Lung fibrosis	136 (26.4%)	90 (23.7%)	46 (34.1%)	.019
Arrhythmia	34 (6.63%)	6 (1.6%)	28 (20.7%)	<.0001
Congestive cardiac failure	6 (1.2%)	4 (1.02%)	2 (1.5%)	.655
Acute myocardial infarction	5 (0.97%)	2 (0.5%)	3 (2.2%)	.116
Hypotension	105 (20.4%)	14 (3.7%)	91 (67.4%)	<.0001
Thrombocytopenia	59 (11.5%)	11 (2.9%)	48 (35.6%)	<.0001
Bleeding episodes	14 (2.7%)	6 (1.6%)	8 (6%)	.008
Deep vein thrombosis	1 (0.19%)	0	1 (0.76%)	N/A
Pulmonary embolism	1 (0.19%)	0	1 (0.76%)	N/A
Thrombosis of abdominal vessels	2 (0.4%)	0	2 (1.5%)	N/A
Intestinal perforation	2 (0.4%)	0	2 (1.5%)	N/A
HAP/VAP	11 (2.1%)	1 (0.3%)	10 (7.6%)	<.0001
BSI Bacterial	11 (2.1%)	5 (1.3%)	6 (4.6%)	.031
BSI Fungal	3 (0.58%)	2 (0.5%)	1 (0.8%)	>.999
Septic shock	58 (11.3%)	4 (1.1%)	54 (40%)	<.0001
Encephalopathy	44 (8.5%)	10 (2.6%)	34 (25.2%)	<.0001
Stroke	5 (0.97%)	1 (0.3%)	4 (3%)	.018
Guillaine Barre syndrome	1 (0.2%)	1 (0.3%)	0	N/A
Acute kidney injury	43 (8.3%)	30 (7.7%)	13 (9.8%)	.539
Hepatitis	53 (10.3%)	37 (9.7%)	16 (11.4%)	.700

Arrhythmia: atrial fibrillation, atrial flutter, ventricular tachycardia, and ventricular fibrillation; HAP/VAP: Hospital associated pneumonia/ Ventilator associated pneumonia; BSI Bacterial: Blood stream bacterial infection; BSI fungal: Bloodstream fungal infection. N/A: Not applicable. p value in bold indicates statistically significant value.

### 4.4 Mortality data

**Figure 1:**
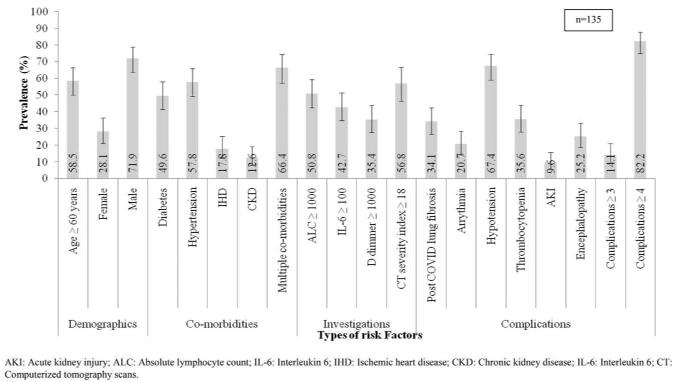
Prevalence of risk factors among COVID-19 patients who died after Tocilizumab and Steroid administration. AKI = Acute kidney injury, ALC = Absolute lymphocyte count, IL-6 = Interleukin 6, IHD = Ischemic heart disease, CKD = Chronic kidney disease, IL-6 = Interleukin 6, CT = Computerized tomography scans.

There were 135 deaths (26.2%) during hospital admission. ICU mortality rate was 40.4% while mortality rate amongst patients requiring NIV/IMV was 55.8%. Of the 71 patients already on NIV or IMV prior to TCZ administration, 70.4% (50/71) died while 29.6% (21/71) survived and were discharged from hospital. Out of the 171 patients who required NIV and/or IMV after TCZ administration, 49.7% (85/171) died while 50.3% (86/171) recovered. Commonest cause of death was acute respiratory distress syndrome leading to respiratory failure. The relative risk (RR) of death was significantly higher in patients with age ≥ 60 years (RR = 1.555 (95% CI: 1.283, 1.884)), concomitant hypertension (RR = 1.284 (95% CI: 1.070, 1.540)), preexisting chronic kidney disease (RR = 2.081 (95% CI: 1.147, 3.773)), IL-6 ≥ 100 pg/ml (RR = 1.515 (95% CI: 1.172, 1.956)), D-dimer ≥ 1000 ng/ml (RR = 2.134 (95% CI: 1.542, 2.954)) and CT severity index ≥ 18 (RR = 2.491 (95% CI: 1.882, 3.299)). Development of systemic complications like lung fibrosis (RR = 1.439 (95% CI: 1.07, 1.934)), cardiac arrhythmia (RR = 13.136 (95% CI: 5.561, 31.029)), hypotension (RR = 18.296 (95% CI: 10.798, 31.000)), new onset or worsening thrombocytopenia (RR = 12.283 (95% CI: 6.574, 22.949)) and encephalopathy (RR = 9.570 (95% CI: 4.862, 18.837)) were also associated with increased risk of death (Figure 1, Table 5).

**Table 5:** Risk factors associated with death in patients administered Tocilizumab and steroids.

Factors	*P* value	Relative risk
Age ≥ 60 years (Ref: < 60 years)	<.0001	1.555 (1.283, 1.884)
Males (Ref: Females)	.226	0.932 (0.827, 1.05)
Co-morbidities		
Diabetes (Ref: No)	.255	1.129 (0.921, 1.385)
Hypertension (Ref: No)	.011	1.284 (1.07, 1.54)
Ischemic heart disease (Ref: No)	.099	1.469 (0.934, 2.31)
Chronic kidney disease (Ref: No)	.015	2.081 (1.147, 3.773)
Multiple co-morbidities (Ref: Single)	.770	1.024 (0.874, 1.199)
Investigations		
ALC < 1000 cells/mm^3^ (Ref: ≥ 1000)	.104	0.861 (0.713, 1.04)
IL-6 ≥ 100 pg/ml (Ref: < 100)	.002	1.515 (1.172, 1.956)
D- dimer ≥ 1000 ng/ml (Ref: < 1000)	<.0001	2.134 (1.542, 2.954)
CT severity index ≥ 18 (Ref: < 18)	<.0001	2.491 (1.882, 3.299)
Systemic complications		
Lung fibrosis (Ref: No)	.019	1.439 (1.07, 1.934)
Arrhythmia (Ref: No)	<.0001	13.136 (5.561, 31.029)
Hypotension (Ref: No)	<.0001	18.296 (10.798, 31)
Thrombocytopenia (Ref: No)	<.0001	12.283 (6.574, 22.949)
Hepatitis (Ref: No)	.511	1.217 (0.7, 2.115)
Acute kidney injury (Ref: No)	.531	1.22 (0.656, 2.268)
Encephalopathy (Ref: No)	<.0001	9.57 (4.862, 18.837)
Complications ≥ 3 (Ref: <3)	<.0001	2.273 (2.012, 2.567)
Complications ≥ 4 (Ref: <4)	<.0001	5.579 (4.327, 7.195)

ALC = Absolute lymphocyte count, IL-6 = Interleukin 6, IHD = Ischemic heart disease, CKD = Chronic kidney disease, IL-6 = Interleukin 6, CT = Computerized tomography scans, Ref = Reference category.

### 4.5 Delayed complications

Out of the 380 patients who recovered and were discharged, 72 (18.9%) needed short term home oxygen therapy after hospital discharge. Of these, two patients reported worsening of respiratory symptoms leading to respiratory failure and death, while 70 patients could be weaned off short term oxygen therapy. Four patients developed late infectious complications (multidermatomal herpes zoster, herpes zoster opthalmicus, acute bacterial cholecystitis and *Escherichia coli* (*E. coli*) bacteremia and septic shock) within 1 month of discharge from hospital. Three patients recovered after treatment, but the patient having *E. coli* bacteremia progressed to septic shock, ARDS and died. One patient developed midbrain encephalitis, 27 days after discharge from hospital and died (Total number of deaths: 139).

## 5 Discussion

The goal of this retrospective observational cohort study conducted at a tertiary level, private hospital in Pune, India was to assess efficacy and safety of combination therapy of TCZ and steroids in tackling CRS developing in patients with severe COVID-19. Our cohort consisted of a relatively elderly population (43% patients ≥ 60 years of age) with pre-existing co-morbidities (72% having co-morbidities) who developed pneumonia, hyperinflammation and ARDS (100% patients having increased inflammatory markers, 62.5% having CT severity score ≥ 15 and 83% having PaO2/FiO2 < 200). 63% patients in our cohort required ICU care while 47% patients needed noninvasive or invasive ventilation to maintain oxygenation. In such a cohort of severely ill patients, administration of TCZ and steroid resulted in clinical improvement in 74% patients, while 26% died due to respiratory failure. The response to TCZ was rapid and sustained in majority of patients requiring supplemental oxygen. Almost 44% patients could also be weaned from ventilator support. However, there was a subset of patients on mechanical ventilation who did show initial improvement after TCZ but subsequently had clinical deterioration and died. This cohort includes 91% of all ICU admissions due to COVID-19 and 96% of all patients receiving mechanical ventilation at NHRC during the said period. To the best of our knowledge, our single center cohort is the largest reported database of patients who were administered TCZ and steroids for management of COVID-19 induced CRS.

Two retrospective cohort studies on TCZ usage from India have been published till date. The strengths of our study compared to the earlier studies include the large number of enrolled patients,^[64]^ strict inclusion and exclusion criteria applied while administering TCZ,^[64,65]^ availability of data about ventilatory outcomes,^[64,65]^ availability of data about systemic complications (including infectious complications)^[64,65]^ and lower mortality rate.^[65]^

In our cohort, 37% patients could be managed in isolation wards without need for intensive care. In an ideal scenario, all patients were candidates for ICU care, but overburdened healthcare system and shortage of ICU beds and ventilators meant that they were managed in wards. In addition, 53% patients required only supplemental oxygen before recovery and did not progress to mechanical ventilation. As per the CORIMUNO-19 RCT, use of TCZ among patients on supplemental oxygen reduced the need for intensive care, noninvasive or invasive mechanical ventilation by almost 40%. The effect of TCZ was numerically higher if combined with steroids.^[61]^ According to the EMPACTA RCT^[62]^ and the TESEO cohort,^[32]^ the likelihood of progression to mechanical ventilation was significantly lower among patients who received TCZ plus standard care than among those who received placebo plus standard care. Reduction in need of ICU care can reduce the risk of long-term complications including death and improve health-related quality of life. Preventing progression to mechanical ventilation greatly alters patient outcomes and leads to better utilization of scarce healthcare resources.

Mortality rate in our cohort was 26%, while mortality rate amongst patients requiring ICU was 40%. Overall mortality rate was similar to that seen in multiple retrospective cohort studies.^[35,36,54,57,64]^ Mortality rate amongst patients requiring mechanical ventilation (NIV/IMV) was 56%. This was higher as compared to other cohort studies.^[40]^ It could be related to delayed intubation and delayed invasive mechanical ventilation policy followed at our institute. Out of 130 patients requiring invasive mechanical ventilation, only 3 could be successfully extubated. On relative risk estimation, age ≥ 60 years, presence of multiple co-morbidities, increased baseline inflammatory markers (IL-6 and D-dimer), high CT severity index and development of systemic complications like lung fibrosis, arrhythmia, encephalopathy, thrombocytopenia and hypotension were significant risk factors associated with death. Females had a higher mortality rate than males in our cohort (30.4% versus 24.9%, Table 5). Early evidence indicates that males have higher overall burden, but females have a higher relative-risk of COVID-19 mortality in India.^[73]^ Marked sex differences in access to health services, with women being less likely to be admitted to hospital than men might result in more severe cases of COVID-19 among women than men in hospital settings and higher mortality.^[74]^

TCZ and steroid combination therapy was safe and well tolerated. Infectious complications like confirmed bacterial and fungal infections (including HAP/VAP and blood stream infections) were seen in 2% patients in the cohort. Low incidence of infectious complications could be because of adequate, prophylactic antibiotic and antifungal therapy prescribed to patients. Meropenem, Teicoplanin and Fluconazole were the commonest antibiotic and antifungal agents prescribed to patients. Transaminitis or hepatitis, which is an adverse event associated with TCZ, was seen in approximately 10% of patients. However, in view of prescription of multiple drugs like Remdesvir, Fluconazole, Doxycycline, Pirfenidone and Nintedanib along with TCZ, it was difficult to identify the exact causative agent of drug induced liver injury. Pulmonary (progression of lung fibrosis) and infectious complications were noted even after discharge from hospital. As a result, close follow up of patients for a period of 3-months after hospital discharge is essential for immediate identification of delayed complications.

### 5.1 Limitations

Our study has several limitations. First, it is not a randomized controlled trial, and therefore unmeasured confounding cannot be ruled out. Second, as for all retrospective studies, some individuals administered TCZ and steroids may be unreported leading to measurement bias and overestimation of safety and efficacy of the combination therapy. Third, a comparator arm was not possible in this pandemic setting. Considering the unavailability of observational or RCT data about efficacy of other regimens to tackle hyperinflammation, worsening of respiratory parameters seen in patients despite receiving potent steroids and life threatening nature of the disease characterized by sudden worsening and rapid progression to respiratory failure over few hours, a comparative arm could not be justified. Fourth, concomitant therapies like antiviral drugs, convalescent plasma, anti-fibrotic agents, supplemental oxygen and ventilation strategies can help in reducing disease severity and clinical improvement. The authors acknowledge the fact that individual contribution of each drug is difficult to estimate. However, considering the predefined criteria for introduction of each drug in patient management, strict inclusion and exclusion criteria applied while administering TCZ and steroids, large number of patients enrolled and improvement in symptoms and oxygenation after TCZ administration, the authors are reasonably confident that this study reliably captures the efficacy of this combination. Fifth, an overwhelmed health care system, inadequate workforce and lack of exhaustive reporting could be responsible for underestimation of co-morbidities, presenting symptoms and complications amongst patients in our cohort. Sixth, Body mass index (BMI) could not be calculated for patients who were bed ridden or those requiring mechanical ventilation. Seventh, CT severity index and PaO2/FiO2 ratio was not available for all patients in our cohort. Eighth, Sequential organ failure assessment (SOFA) score was not performed in patients admitted in ICU.^[75]^ Ninth, ventilator parameters like positive end expiratory pressure (PEEP) and plateau pressure were not available for all patients started on NIV or IMV. Tenth, patients were followed up after discharge from hospital for 1 month. As a result, long term complications due to immunomodulatory therapy could not be identified.

Despite these limitations, this retrospective cohort study from Western India adds to the growing body of literature on use of TCZ and steroids as an anti-inflammatory combination therapy in tackling cytokine release syndrome and resultant ARDS in COVID-19.

## 6 Conclusions

Combination therapy of TCZ and steroids is likely to be a safe and effective treatment modality in management of COVID-19 associated cytokine release syndrome. Efficacy of this anti-inflammatory combination therapy needs to be validated in large randomized controlled clinical trials.

## Acknowledgments

Manisha Ghate MD, PhD (National AIDS Research Institute (NARI), Pune, India) - edited the manuscript.

## Author contributions

**Conceptualization:** Ameet N Dravid, Reema Kashiva, Zafer Khan, Prashant Potdar, Milind Mane, Rakesh Borse, Uma Mahajan, Dileep Mane.

**Data curation:** Ameet N Dravid, Reema Kashiva, Zafer Khan, Aparna Kodre, Prashant Potdar, Milind Mane, Rakesh Borse, Dattatraya Patil, Debashis Banerjee, Kailas Bhoite, Reshma Pharande, Suraj Kalyani, Prathamesh Raut, Madhura Bapte, Anshul Mehta, M Sateesh Reddy, Krushnadas Bhayani, S S Laxmi, P D Vishnu, Shipra Srivastava, Shubham Khandelwal, Sailee More, Rohit Shinde, Mohit Pawar, Dileep Mane.

**Formal analysis:** Ameet N Dravid, Uma Mahajan, Gaurav Joshi.

**Investigation:** Ameet N Dravid, Reema Kashiva, Zafer Khan, Danish Memon, Aparna Kodre, Prashant Potdar, Milind Mane, Rakesh Borse, Vishal Pawar, Dattatraya Patil, Kailas Bhoite, Reshma Pharande, Anshul Mehta, Krushnadas Bhayani, S S Laxmi, Amol Harshe, Sagar Kadam.

**Methodology:** Ameet N Dravid, Zafer Khan, Danish Memon, Aparna Kodre, Prashant Potdar, Milind Mane, Rakesh Borse, Vishal Pawar, Dattatraya Patil, Kailas Bhoite, Reshma Pharande, Amol Harshe, Sagar Kadam, Uma Mahajan, Dileep Mane.

**Project administration:** Ameet N Dravid, Reema Kashiva, Zafer Khan, Danish Memon, Aparna Kodre, Milind Mane, Rakesh Borse, Vishal Pawar, Dattatraya Patil, Debashis Banerjee, Kailas Bhoite, Reshma Pharande, Suraj Kalyani, Prathamesh Raut, Anshul Mehta, M Sateesh Reddy, S S Laxmi, P D Vishnu, Dileep Mane.

**Resources:** Ameet N Dravid, Danish Memon, Aparna Kodre, Prashant Potdar.

**Software:** Ameet N Dravid, Prashant Potdar.

**Supervision:** Ameet N Dravid, Reema Kashiva, Zafer Khan, Danish Memon, Aparna Kodre, Prashant Potdar, Rakesh Borse, Vishal Pawar, Dattatraya Patil, Debashis Banerjee, Kailas Bhoite, Reshma Pharande, Suraj Kalyani, Prathamesh Raut, Madhura Bapte, Anshul Mehta, M Sateesh Reddy, Krushnadas Bhayani, S S Laxmi, P D Vishnu, Dileep Mane.

**Validation:** Ameet N Dravid, Reema Kashiva, Zafer Khan, Danish Memon, Prashant Potdar, Vishal Pawar, Debashis Banerjee, Dileep Mane.

**Visualization:** Ameet N Dravid, Reema Kashiva, Zafer Khan, Danish Memon, Prashant Potdar.

**Writing – original draft:** Ameet N Dravid, Uma Mahajan, Gaurav Joshi, Dileep Mane.

**Writing – review & editing:** Ameet N Dravid, Uma Mahajan, Gaurav Joshi, Dileep Mane.
